# Accuracy of Computerized Optical Impression Making in Fabrication of Removable Dentures for Partially Edentulous Jaws: An In Vivo Feasibility Study

**DOI:** 10.3390/jfb14090458

**Published:** 2023-09-05

**Authors:** Babak Saravi, Julia Ilbertz, Kirstin Vach, Ralf J. Kohal, Sebastian B. M. Patzelt

**Affiliations:** 1 Department of Orthopedics and Trauma Surgery, Medical Center—University of Freiburg, Faculty of Medicine, University of Freiburg, Hugstetter Street 55, 79106 Freiburg, Germany; babak.saravi@jupiter.uni-freiburg.de; 2 Department of Anesthesiology, Perioperative and Pain Medicine, Brigham and Women’s Hospital, Harvard Medical School, Boston, MA 02215, USA; 3 Department of Prosthetic Dentistry, Medical Center—University of Freiburg, Center for Dental Medicine, Faculty of Medicine, University of Freiburg, Hugstetter Street 55, 79106 Freiburg, Germany; julia.ilbertz93@gmail.com (J.I.); ralf.kohal@uniklinik-freiburg.de (R.J.K.); 4 Private Dental Clinic, Am Dorfplatz 3, 78658 Zimmern ob Rottweil, Germany; 5 Institute of Medical Biometry and Statistics, Medical Center—University of Freiburg, Faculty of Medicine, University of Freiburg, Stefan-Meier-Str. 26, 79104 Freiburg, Germany; kirstin.vach@uniklinik-freiburg.de

**Keywords:** intraoral scan, conventional impression, digital impression, partially edentulous, dental prosthesis, 3D analyses

## Abstract

The use of computerized optical impression making (COIM) for the fabrication of removable dentures for partially edentulous jaws is a rising trend in dental prosthetics. However, the accuracy of this method compared with that of traditional impression-making techniques remains uncertain. We therefore decided to evaluate the accuracy of COIM in the context of partially edentulous jaws in an in vivo setting. Twelve partially edentulous patients with different Kennedy classes underwent both a conventional impression (CI) and a computerized optical impression (COI) procedure. The CI was then digitized and compared with the COI data using 3D analysis software. Four different comparison situations were assessed: Whole Jaw (WJ), Mucosa with Residual Teeth (M_RT), Isolated Mucosa (IM), and Isolated Abutment Teeth (AT). Statistical analyses were conducted to evaluate group differences by quantifying the deviation values between the CIs and COIs. The mean deviations between the COIs and CIs varied significantly across the different comparison situations, with mucosal areas showing higher deviations than dental hard tissue. However, no statistically significant difference was found between the maxilla and mandible. Although COIM offers a no-pressure impression method that captures surfaces without irritation, it was found to capture mucosa less accurately than dental hard tissue. This discrepancy can likely be attributed to software algorithms that automatically filter out mobile tissues. Clinically, these findings suggest that caution is required when using COIM for prosthetics involving mucosal tissues as deviations could compromise the fit and longevity of the prosthetic appliance. Further research is warranted to assess the clinical relevance of these deviations.

## 1. Introduction

The field of dentistry has continually evolved in terms of its methods for capturing intraoral impressions, which serve as foundational steps for various dental treatments. Two principal methods have gained prominence: conventional impressions (CIs) and computerized optical impressions (COIs) [1].

Conventional impressions involve creating a physical model through the use of dental impression materials. This model can then be used in two different workflows: it may either facilitate the direct fabrication of dental restorations (known as the conventional workflow) or undergo digital scanning to enable computer-aided designing and manufacturing (referred to as the hybrid workflow).

On the other side of the spectrum, COIs enable a leap into a fully digital paradigm. With the aid of intraoral scanners, COIs enable an end-to-end digital workflow [2,3]. This not only enables the immediate commencement of computer-aided processes, but it also offers several advantages, such as material, time, and cost savings [4]. Additionally, COIs circumvent the potential pitfalls associated with the conventional workflow, such as dimensional changes resulting from impression material deficiencies, processing or storage errors, and other errors that occur along the process chain [2]. Furthermore, intraoral scanners offer additional functionalities, such as shade determination, caries diagnosis, and preparation analysis.

Interestingly, the choice between these two methods is not made uniformly across practitioners. The transition from conventional to computerized optical impression making (COIM) requires a learning curve; training significantly influences the accuracy and scanning time of complete-arch scans [5]. Notably, impression method preferences vary, with experienced dentists often favoring conventional impressions, while students lean towards digital impressions [6]. Further, despite the high acquisition costs and system incompatibilities, patients often report higher satisfaction with digital impressions, particularly those with gag reflexes, breathing difficulties, or taste sensitivities [7].

While COIM has shown promise for single crowns and short-span dentures [8,9], a considerable gap exists in the literature concerning its applicability to more complex restorative scenarios, specifically tooth-supported, jaw-spanning partial dentures. Notably, most existing studies focus on fully dentate models or patients with defect-free teeth, often in in vitro settings [10,11,12,13,14,15]. This has led to a limited understanding of how these impression methods perform in real-world situations involving partially edentulous jaws.

Given this backdrop, the current study aims to address this significant gap in the existing research by focusing on the practical feasibility of capturing impressions of partially edentulous jaws using COIM in an in vivo setting. This study introduces the hypothesis that COIM will demonstrate accuracy levels comparable to those of CIs in clinical scenarios involving partially edentulous jaws.

## 2. Materials and Methods

### 2.1. Participants

**Figure 1:**
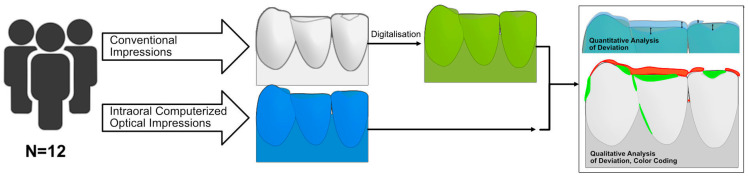
Schematic representation of the study design.

A total of 30 participants were recruited from the Department of Prosthetic Dentistry at the University Hospital Freiburg. The participants were partially dentate patients who had been provided with a removable partial denture for the upper and/or lower jaw. These were either double crowns or model cast restorations. Participation was voluntary and could be withdrawn at any time. All procedures involving human participants were performed in accordance with the ethical standards of the institutional research committee (approval number: 343/15) and with the 1964 Helsinki Declaration and its later amendments, or comparable ethical standards. All participants gave their informed consent. No additional financial compensation was provided to the patients. The inclusion criteria were as follows: patients had to be over 18 years of age and have at least one partially dentate jaw with a minimum of two remaining abutment teeth. Exclusion criteria included any disease or condition that precluded treatment (infectious diseases, pregnancy, etc.) and lack of a signed consent form. The study design is illustrated in Figure 1 and will be elaborated in detail in the subsequent sections.

### 2.2. Conventional Impressions

Individual custom impression trays were utilized for the conventional impressions, which were made using multifunctional acrylates. The abutment teeth were anesthetized and relative isolation was ensured through the use of a lip and cheek retractor, air pressure, saliva ejectors, and cotton rolls. Each impression was taken a week after abutment teeth preparation using a double mix impression technique with vinyl siloxane ether material (Identium light and heavy, Kettenbach GmbH & Co. KG, Eschenburg, Germany) under relative isolation. After gingival retraction, a low-viscosity material was injected around the prepared teeth while the custom tray was filled with a high-viscosity material. The tray was then removed and disinfected, and type IV super hard plaster (Fujirock EP Classic, GC Corporation, Tokyo, Japan) was poured into it by a dental technician, in accordance with the manufacturer’s processing and setting times. The models were examined, trimmed, and digitally scanned with an optical lab scanner (D2000, 3Shape, Copenhagen, Denmark, Software 3Shape Version 2015), and the data were saved as STL files. The measurements of the models were performed by S.P., who has over 10 years of experience in intraoral scanning and conventional impression techniques.

### 2.3. Intraoral Computerized Optical Impression

**Figure 2:**
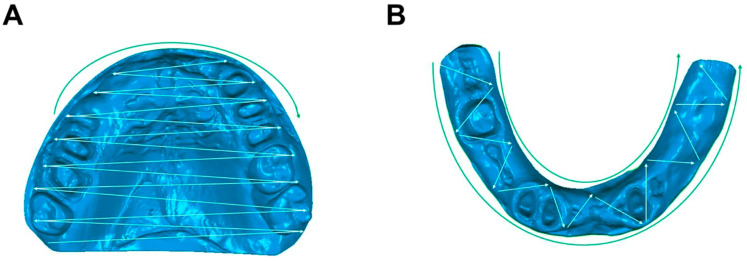
Schematic representation of the scan path in the (**A**) upper and (**B**) lower jaw.

COIM was performed using a commercially available intraoral scanner (True Definition Scanner, 3M, St. Paul, MN, USA, Software version 5.0.2-production-eu) via the application of a titanium dioxide (High-Resolution Scanning Spray, 3M, St. Paul, MN, USA) scanning powder applied using a battery-powered powder applicator. The True Definition Scanner uses an active wavefront sampling technique and captures data in real-time at a capture speed of 20 3D images per second. COIM was always performed directly after the conventional impression to obtain a second impression. Impressions of the partially edentulous patients’ upper and lower jaws were obtained under relative isolation. The abutment teeth were anesthetized and prepared using the double cord technique before they were dusted with scanning powder. A standardized scan path was adhered to (Figure 2), and the abutment teeth were digitally isolated from the scan file. For the upper jaw, scanning proceeded from the back right to the front left, in a zigzag pattern, with missing areas added subsequently (Figure 2A). For the lower jaw, scanning proceeded from the back right to the front left in a zigzag pattern along the jaw ridge, and the vestibule and sublingual areas were then scanned (Figure 2B). Missing areas were added subsequently. The intraoral scans were performed by S.P., who is extensively trained and has over 10 years of experience in intraoral scanning procedures.

### 2.4. Indirect Extraoral Digitization and Processing of Models

Master models were scanned using an optical laboratory scanner (D2000, 3Shape). This lab scanner operates on the principle of structured light projection via a multiline blue LED and utilizes four 5.0-megapixel cameras. The manufacturer claims that it has an accuracy of 5 μm (according to ISO 12836) [16]. The scanning time for a complete dental arch was approximately 16 seconds. The abutment teeth scans were fully captured and auto-aligned. The scan files were uploaded to a proprietary portal and downloaded as STL files.

**Figure 3:**
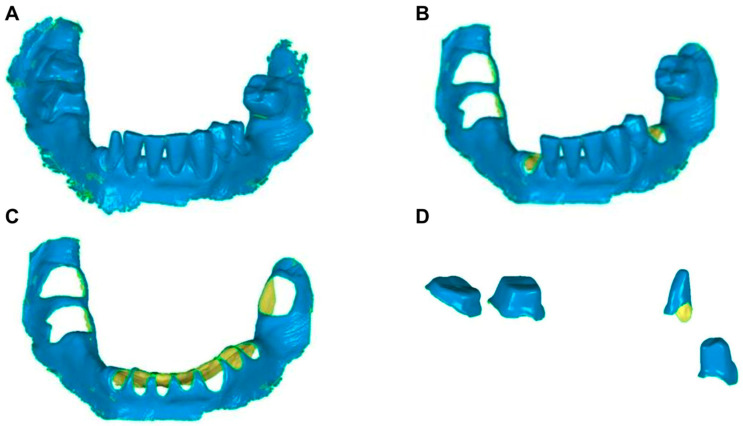
Illustration of the different cuts (TrueDefinition model). (**A**) Whole Jaw (WJ); (**B**) Mucosa with Residual Teeth (M_RT); (**C**): Isolated Mucosa (IM); and (**D**): Isolated Abutment Teeth (AT).

Several parameters were uniformly preset in the 3D evaluation software (Geomagic Control 2014, software version 2014.0.1.1671, Geomagic, Morrisville, NY, USA). The master and TrueDefinition datasets (obtained via COIM) were trimmed to eliminate plaster parts and artifacts, respectively. Four different comparison situations were assessed for each patient: Whole Jaw (WJ), Mucosa with Residual Teeth (M_RT), Isolated Mucosa (IM), and Isolated Abutment Teeth (AT; Figure 3). Each situation involved different trimming and selection processes. In all cases, the cutting tool was carefully used to maintain model integrity. After processing, the individual models of each patient were combined and saved as WRP files, a proprietary 3D modeling format used by Geomagic. Using the 3D evaluation software, the scans of the master models were virtually superimposed (best-fit algorithm) and compared (3D comparisons) with the intraoral scans in the next step.

### 2.5. Superimposition and 3D Comparison

Following model preparation, corresponding segments from the COIs and CIs were superimposed using Geomagic Control 2014’s Best-Fit-Alignment algorithm. This process, known as registration, utilizes the software’s Best-Fit method to obtain the maximum congruence of the datasets to be compared. Parameters such as sample size and tolerance value were preset in the software, and registration was executed automatically. A 3D comparison was then performed, providing both quantitative and qualitative analyses. The quantitative assessment included maximum and minimum deviations, average positive and negative deviations, root mean squares, and standard deviations between the digitized and digital models. The qualitative analysis involved a color-coded image illustrating the deviation distribution between the reference and digital models. The areas marked in green showed no deviations. The areas that changed into the positive range were colored from yellow to orange to red, and the areas that moved into the negative range were colored from light blue to dark blue. The settings for Whole Jaw (WJ), Mucosa with Residual Teeth (M_RT), Isolated Mucosa (IM), and Isolated Abutment Teeth (AT) were slightly modified for each patient due to greater variability. The tolerance value was increased, the sample size was broadened for a more accurate fit, and the spectrum for color coding was expanded.

### 2.6. Statistical Analyses

The collected data were tabulated using spreadsheet software (Microsoft Excel for Microsoft 365, Version 2111, Microsoft, Redmond, Washington, DC, USA). The maximum and minimum average deviations, the mean values, and the standard deviations (SDs) (in μm) were included in the table. The statistical evaluation was carried out with a statistics program (STATA, Version 16.1, StataCorp LLC, College Drive, TX, USA). Descriptive statistics were obtained by calculating the medians and mean values ± standard deviations. A linear mixed regression model was used to evaluate both the dependence of the mean deviation on the used sections and to distinguish the influence of the number of abutment teeth on the mean deviation within the subgroups. To solve the problem of multiple testing in several pairwise comparisons, Scheffé’s method was used to adjust the *p*-values. Box plots were created for the graphical representation of the data. A *p*-value < 0.05 was considered significant.

## 3. Results

### 3.1. Enrollment

During the recruitment period (October 2015—July 2016), 24 individuals expressed their interest in participating in the study. However, only 12 patients met the inclusion criteria and were finally enrolled. Out of the initially considered 24 participants, 12 withdrew their participation after being informed about the study’s content, or because they did not need prosthetic treatment, as per the inclusion criteria. Among the final 12 participants, 8 received prosthetic treatment in the lower jaw, 3 received prosthetic treatment in the upper jaw, and 1 received prosthetic treatment in both jaws. Consequently, 13 conventional impressions and 13 computer-aided optical impressions were taken. A total of 48 3D comparisons were made (Table 1). Due to the insufficient number of identical abutment teeth, individual abutment teeth were not compared statistically.

### 3.2. Comparisons across Different Sections

**Figure 4:**
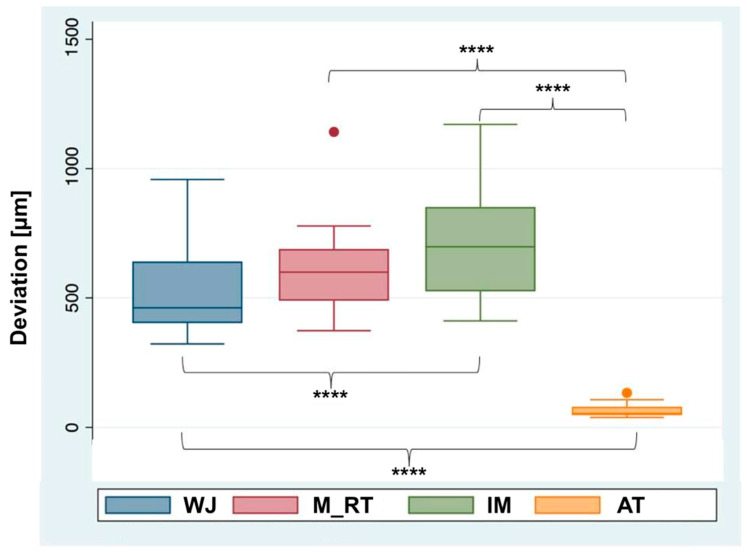
Boxplot comparison of mean deviations of different sections. WJ = Whole Jaw, M_RT = Mucosa with Residual Teeth, IM = Isolated Mucosa, and AT = Isolated Abutment Teeth. Circles indicate outliers. **** *p* < 0.0001.

The descriptive statistical evaluation revealed that the smallest mean deviations were in the AT sections. The largest mean deviations were found in the IM sections, with the WJ and M_RT sections lying in between (Figure 4). The 13 WJ sections resulted in a median of 461.5 μm and a mean of 542.3 ± 189.5 μm. For the nine M_RT sections, a median of 599.5 μm and a mean of 633.4 ± 230.2 μm were found. For the 13 IM sections, a median of 697.5 μm and a mean of 736.8 ± 247.7 μm were calculated. For the 13 AT sections, a median of 53.5 μm and a mean of 67.3 ± 28.8 μm were found (Table 2).

### 3.3. Comparisons across Different Sections Stratified by Jaw

**Figure 5:**
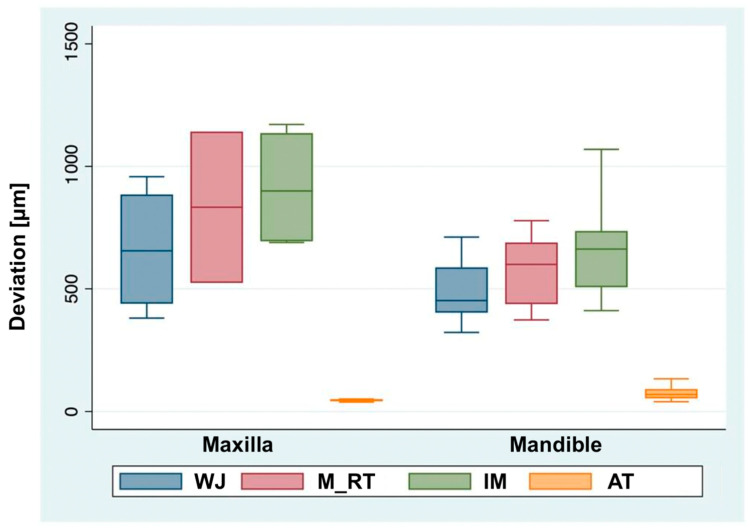
Boxplot comparison of mean deviations of different sections stratified by jaw. WJ = Whole Jaw, M_RT = Mucosa with Residual Teeth, IM = Isolated Mucosa, and AT = Isolated Abutment Teeth.

When they were divided according to maxilla and mandible, a similar pattern emerged for the individual sections as that presented in Section 3.2. The values of the measured mean deviations in the maxilla were greater than those in the mandible, with the exception of the AT sections (Figure 5). However, none of the comparisons revealed any statistical significance. In the maxilla, the four WJ sections yielded a median of 655 μm and a mean of 662.1 (SD ± 268.7) μm. For the two M_RT sections, a median of 833 μm and a mean of 833 (SD ± 437) μm were calculated. For the four IM sections, a median of 899.8 μm and a mean of 914.9 (SD ± 256.7) μm were found. For the four AT sections, the median was 47.3 μm and the mean was 46 (SD ± 5.8) μm. In the mandible, the nine WJ sections yielded a median of 452 μm and a mean of 489 (SD ± 128.2) μm. For the seven M_RT sections, a median of 599.5 μm and a mean of 576.4 (SD ± 147.6) μm were calculated. For the nine IM sections, the median was 662 μm and the mean was 657.6 (SD ± 210.8) μm. For the nine AT sections, a median of 69.5 μm and a mean of 76.8 (SD ± 30.1) μm were found (Table 3). Furthermore, a comparison was made between the maxilla and the mandible across all the sections. The difference between the maxilla and the mandible was 151.6 ± 77.9 μm (*p* = 0.052).

### 3.4. Comparisons across Different Sections Stratified by Abutment Teeth

The deviations for the M_RT and IM sections were higher than those for the WJ sections, although these deviations were not statistically significant. The smallest mean deviations were determined for the AT sections. Finally, the influence of the number of abutment teeth on the mean deviations of the COIs was assessed. There was no evidence of an association for the WJ sections, the M_RT sections, the IM sections, or the AT sections.

### 3.5. Qualitative Analyses

**Figure 6:**
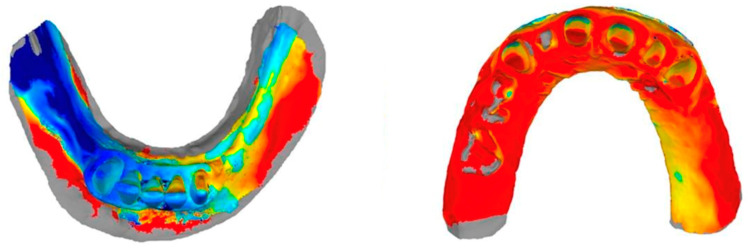
Exemplary color-coded representation of a 3D comparison of the mandible (**left**), which illustrates high deviations and missing data in the sublingual and vestibule areas, and the upper jaw (**right**), for which there is missing data in the larger mucosal groove areas.

**Figure 7:**
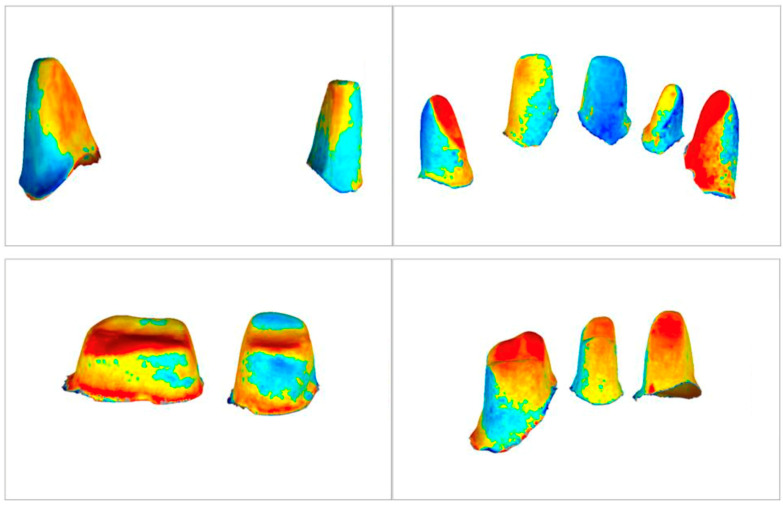
Exemplary color-coded representation of 3D comparisons of four sections of Isolated Abutment Teeth (AT).

A visual analysis of the color-coded images revealed discrepancies in both the horizontal and vertical planes. The highest deviations are represented in dark blue and dark red in the color-coded images, while no or minor deviations are indicated by green, light yellow, and light blue, respectively. The mucosal areas showed the largest deviations. No remarkable differences were revealed between the different sections. High deviations (blue and red) and missing data (gray) were particularly noticeable in the sublingual areas, in the vestibule, and in the larger mucosal grooves (extraction sockets, palatal folds). The interdental papillae between the remaining teeth often showed large deviations. For the WJ sections, most of the deviations were in the light yellow to light blue range, i.e., between +130 μm and −130 μm. For the M_RT and IM sections, there were often larger scatterings. It was not possible to specify an exact μm range within which most of the deviations occurred (Figure 6 and Figure 7).

## 4. Discussion

This in vivo study examined the accuracy of computer-assisted optical impressions of partially edentulous jaws compared with that of conventional impressions. Several studies have reported that COIM offers excellent accuracy when applied to fully edentulous jaws, with deviations ranging from 30 to 108 μm [17,18,19]. However, the present study found significantly higher deviations for the WJ, M_RT, and IM sections than for the AT section. This may be because intraoral scanners are optimized for capturing tooth structure and therefore produce higher deviations in areas with mucosal tissues. The present study also found that the number of abutment teeth did not significantly influence the accuracy of COIM. The visual analysis revealed that the mucosal areas, particularly the sublingual, vestibulum, and interdental regions, exhibited the largest deviations. These findings suggest that mucosal areas pose challenges for intraoral scanners, making it difficult to achieve accurate digital impressions. Our initial hypothesis posited that COIM would offer accuracy levels comparable to those of CIs in clinical scenarios involving partially edentulous jaws. Based on our results, this hypothesis was not fully supported; while COIM exhibited a similar degree of accuracy to that of the CI technique when capturing abutment teeth, significantly higher deviations were noted in mucosal areas.

When comparing our results with those of previous studies, it is important to note the discrepancies in the methodologies and equipment used. The existing literature includes numerous in vitro studies on impressions and scanning accuracy. However, most of these studies focused on fully dentate models [10,17,20] or patients with intact teeth [10,11,12,13,14], which do not adequately represent the typical cases that require treatment. Only a limited number of studies have specifically addressed the accuracy of impressions of partially edentulous jaws [21,22,23,24]. Furthermore, these studies primarily examined defect-free teeth and did not compare their results to those obtained using the current gold standard for complete-arch impressions, the conventional impression [21,22,23,24]. To the best of our knowledge, there are currently no in vivo studies on the accuracy of COIs of partially edentulous jaws, making direct comparisons difficult. In vitro studies have shown varying results. For instance, in the present study, the mean deviations for the IM sections were higher than the values reported by Hack et al., who applied the 3M system (True Definition Scanner and Lava C.O.S) to edentulous jaws [25]. These differences could be attributed to variations in operator experience, different reference scanners, or the use of different impression materials. It should be noted that the findings of the present study may not be directly comparable to those of in vitro studies due to the inability of in vitro studies to fully simulate clinical conditions. Factors such as patient movement, limited mouth opening, and the presence of blood and saliva can impact the accuracy of intraoral scanners. Future studies should therefore validate the present findings in vivo. A general overview of the advantages and disadvantages of COIM and the CI technique is provided in Table 4. Although our focus is on computer-assisted optical impressions, it is worth noting the broader advancements in technology that are applicable to the maxillofacial and dental fields, such as the growth in medical 3D printing, which is used for craniomaxillofacial surgery applications [26]. These innovations indicate a trend towards more precise and customizable treatment options and shed light on the need for ongoing research to optimize existing technologies, such as intraoral scanners. One critical advantage of COIM is its potential role in minimizing the spread of contagious diseases. Unlike conventional impression methods, which require direct physical contact and multiple exchanges of dental materials between clinicians and laboratories, digital impressions can be transferred electronically, thereby reducing the risk of cross-contamination. This is especially vital in times of global pandemics, during which infection control measures are a top priority. The implementation of COIM also offers benefits in terms of minimally invasive dentistry. The precise impressions made through COIM are particularly crucial for cases requiring minimally prepared dental bridges, such as inlay-retained bridges. A study by Tatarciuc et al. (2021) emphasizes that the proper design of such bridges, including the areas of maximum pressure on the supporting teeth, is crucial for their success [27]. Accurate digital impressions enable precise designs and contribute to the longevity of minimally invasive dental restorations.

For the COIM, an intraoral scanner operating on the active wavefront sampling principle was utilized. This scanner, which utilizes video technology and captures 20 frames per second, is suitable for recording smooth surfaces, such as mucosal areas in partially edentulous jaws [25]. The application of titanium dioxide powder generates small surface points that enable the assembly of 3D video recordings. Given its essential role in the active wavefront sampling capture technique, any negative impact on the accuracy is considered minimal. However, it remains unclear whether powder application negatively affects accuracy [25]. Saliva and tongue movements make it difficult to maintain the powder layer, necessitating occasional re-powdering during scanning. In this context, powder-free systems may be easier to use. The 3M True Definition Scanner was selected for this study due to its proven in vitro accuracy and repeatability [28,29,30,31]. Notably, since there is no recommended manufacturer-specific scanning path for partially edentulous jaws, the scanning paths were based on those described in the literature [25,32]. A zigzag-shaped scanning path was utilized, and the abutment teeth were scanned using diffuse movements in all directions until all of the areas were captured, without any gaps. Following this standardized scanning path in all cases ensured consistency and minimized variations due to different scanning paths [33].

Nevertheless, the present study has certain limitations. To achieve a sufficiently high sample size, the study incorporated various patients with differing numbers of remaining teeth. This variability could have impacted the results and contributed to the scatter within the data. Given that this was a feasibility study, and one of the first of its kind, no a priori power analysis was performed to determine the sample size. Consequently, the study may be underpowered for detecting smaller effect sizes. Thus, the results should be interpreted as preliminary and exploratory rather than confirmatory. Nevertheless, despite the lack of formal sample size calculation, we consider our sample size to be in line with or exceeding those found in similar feasibility studies [17,21,34]. Teeth and jaws have individual anatomical variations, and patients differ in salivary flow and mouth opening. These factors could have led to increased scatter in the deviation data. Enhanced salivary flow makes both COIs and conventional impressions more difficult to obtain. However, hybrid impression materials exhibit excellent hydrophilicity, suggesting that enhanced salivary flow has a minimal impact on the accuracy of conventional impressions. Conversely, studies have shown that the presence of saliva affects the accuracy of intraoral scanners [35,36]. Saliva can wash away the applied scan powder, simplifying the detection of wet areas but necessitating additional powder during scanning [36]. In this study, an experienced dentist performed the COIM under relatively dry conditions (i.e., minimal salivary contamination). However, complete prevention was not guaranteed. Importantly, the titanium dioxide powder used in this study had a specific role: it created small dots on the surface that were instrumental for the merging of the 3D video images. Given that our scanning technology operated on the principle of active wavefront sampling, this approach was deemed advantageous, particularly for the acquisition of smooth surfaces, such as mucosal areas in partially edentulous jaws [25]. Furthermore, the plaster models were digitized using an optical laboratory scanner. Although optical laboratory scanners achieve acceptable accuracy, discrepancies between the plaster models and the digitized models can occur [37,38]. The manufacturer of the laboratory scanner used in this study specifies an accuracy of 5 μm (ISO 12836) [16].

Notably, the cropping of the datasets to the analysis area was performed by one individual, who followed the same principle for each patient in order to ensure consistent cropping. Nevertheless, discrepancies in the manual cropping may have occurred due to variations in the selection of the areas. It is also important to note that the accuracy analysis was conducted using only one intraoral scanner, and that the results therefore cannot be generalized to all optical systems, especially to current systems. Additionally, the visual analyses of the upper jaws were challenging due to the absence of a hard palate in the reference models. This limited the accuracy of the visual assessments of the complete hard palates.

From a clinical standpoint, the findings of our study have significant implications for prosthetic treatment decisions concerning partially edentulous patients. The higher deviations observed in the mucosal areas via COIM underscore the need for caution when choosing an impression method for prosthetics involving these tissues. Practitioners should be aware that while COIM may provide excellent results for teeth and hard tissues, its accuracy in mucosal areas may be compromised, potentially leading to ill-fitting prosthetics and subsequent clinical complications. This is particularly pertinent in the design and manufacture of partial dentures, which require precise impressions for a clinically acceptable fit. Our results also call attention to the potential for higher deviations when scanning mucosal tissues, which could affect the longevity and clinical success of the prosthetic appliance. While conventional impressions may remain the preferred method for capturing these areas, our study illuminates the need for technological advancements in intraoral scanners to improve their performance in capturing soft tissue details.

## 5. Conclusions

This study offers the first in vivo evaluation of the accuracy of COIs of partially edentulous jaws compared with CIs. Our findings reveal that while COIM shows high accuracy when capturing hard tissues such as abutment teeth, it exhibits greater deviations in mobile mucosal areas, underscoring its limitations when capturing soft tissues. From a clinical perspective, this is a crucial consideration for dental practitioners when selecting impression techniques for prosthetic applications involving soft tissues. While COIM may offer certain advantages, its shortcomings when capturing mucosal areas cannot be ignored and may result in ill-fitting prosthetics and potential clinical complications. Thus, our data suggest that conventional impressions may currently be more reliable for capturing complex soft tissue details in partially edentulous jaws. It is essential to acknowledge the limitations of our study. We used a single type of intraoral scanner, and the sample patients had varying numbers of remaining teeth, which might have introduced variability. Additionally, our study did not account for factors such as patient movement and saliva flow, which could potentially have influenced the accuracy of both the COIs and the CIs. Future research should focus not only on evaluating contemporary intraoral scanners, but also on studying how variables such as patient movement and saliva flow could impact accuracy. Our work serves as a foundational study in this research avenue, setting the stage for further in vivo investigations to refine and validate these findings.

## Figures and Tables

**Table 1:** Number of comparisons stratified by section, jaw, and abutment teeth.

**Jaw**	**WJ (n = 13)**	**M_RT** **(n = 9)**	**IM (n = 13)**	**AT (n = 13)**
Maxilla	4	2	4	4
Mandible	9	7	9	9
**Abutment teeth**	**WJ (n = 13)**	**M_RT** **(n = 9)**	**IM (n = 13)**	**AT (n = 13)**
2	4	4	4	4
3	3	2	3	3
4	4	2	4	4
5	2	1	2	2

Note: WJ = Whole Jaw, M_RT = Mucosa with Residual Teeth, IM = Isolated Mucosa, and AT = Isolated Abutment Teeth.

**Table 2:** Descriptive statistics for each section.

Section	Median (μm)	Mean (μm)	SD (μm)
WJ	461.5	542.3	189.5
M_RT	599.5	633.4	230.2
IM	697.5	736.8	247.7
AT	53.5	67.3	28.8

Note: WJ = Whole Jaw, M_RT = Mucosa with Residual Teeth, IM = Isolated Mucosa, and AT = Isolated Abutment Teeth.

**Table 3:** Descriptive statistics for each section stratified by jaw.

Jaw	Section	Median (μm)	Mean (μm)	SD (μm)
Maxilla	WJ	655	662.1	268.7
Maxilla	M_RT	833	833	437
Maxilla	IM	899.8	914.9	256.7
Maxilla	AT	47.3	46	5.8
Mandible	WJ	452	489	128.2
Mandible	M_RT	599.5	576.4	147.6
Mandible	IM	662	657.6	210.8
Mandible	AT	69.5	76.8	30.1

Note: WJ = Whole Jaw, M_RT = Mucosa with Residual Teeth, IM = Isolated Mucosa, and AT = Isolated Abutment Teeth.

**Table 4:** Advantages and disadvantages of computerized optical impression making (COIM) and conventional impressions (CIs).

Criteria	COIM	CIs
Accuracy	High precision, but this may vary with operator skill	Dependent on material and technique
Time-Efficiency	Generally quicker	May be time-consuming
Patient Comfort	Non-invasive, more comfortable	May be uncomfortable due to materials
Cost	Higher initial investment	Generally lower cost
Skill Level	Requires technical proficiency	Requires traditional expertise
Portability	Digital information is transferable	Transport of material required
Error Recovery	Easier to correct mistakes digitally	Errors often require reworking

## Data Availability

The data presented in this study are available on request from the corresponding author.
